# The NILS Study Protocol: A Retrospective Validation Study of an Artificial Neural Network Based Preoperative Decision-Making Tool for Noninvasive Lymph Node Staging in Women with Primary Breast Cancer (ISRCTN14341750)

**DOI:** 10.3390/diagnostics12030582

**Published:** 2022-02-24

**Authors:** Ida Skarping, Looket Dihge, Pär-Ola Bendahl, Linnea Huss, Julia Ellbrant, Mattias Ohlsson, Lisa Rydén

**Affiliations:** 1 Department of Clinical Sciences, Division of Oncology, Lund University, 221 85 Lund, Sweden; par-ola.bendahl@med.lu.se; 2 Department of Clinical Physiology and Nuclear Medicine, Skåne University Hospital, 221 85 Lund, Sweden; 3 Department of Clinical Sciences, Division of Surgery, Lund University, 221 85 Lund, Sweden; looket.dihge@med.lu.se (L.D.); julia.ellbrant@med.lu.se (J.E.); lisa.ryden@med.lu.se (L.R.); 4 Department of Plastic and Reconstructive Surgery, Skåne University Hospital, 205 02 Malmö, Sweden; 5 Department of Clinical Sciences Lund, Clinical Sciences Helsingborg, Lund University, 221 85 Lund, Sweden; linnea.huss@med.lu.se; 6 Department of Surgery, Helsingborg General Hospital, 252 23 Helsingborg, Sweden; 7 Department of Surgery, Skåne University Hospital, 205 02 Malmö, Sweden; 8 Department of Astronomy and Theoretical Physics, Division of Computational Biology and Biological Physics, Lund University, 221 85 Lund, Sweden; mattias.ohlsson@thep.lu.se

**Keywords:** breast neoplasm, artificial neural network, staging, axilla, lymph nodes, validation study

## Abstract

Newly diagnosed breast cancer (BC) patients with clinical T1–T2 N0 disease undergo sentinel-lymph-node (SLN) biopsy, although most of them have a benign SLN. The pilot noninvasive lymph node staging (NILS) artificial neural network (ANN) model to predict nodal status was published in 2019, showing the potential to identify patients with a low risk of SLN metastasis. The aim of this study is to assess the performance measures of the model after a web-based implementation for the prediction of a healthy SLN in clinically N0 BC patients. This retrospective study was designed to validate the NILS prediction model for SLN status using preoperatively available clinicopathological and radiological data. The model results in an estimated probability of a healthy SLN for each study participant. Our primary endpoint is to report on the performance of the NILS prediction model to distinguish between healthy and metastatic SLNs (N0 vs. N+) and compare the observed and predicted event rates of benign SLNs. After validation, the prediction model may assist medical professionals and BC patients in shared decision making on omitting SLN biopsies in patients predicted to be node-negative by the NILS model. This study was prospectively registered in the ISRCTN registry (identification number: 14341750).

## 1. Introduction

Most breast cancer (BC) patients are diagnosed at an early stage of the disease, and surgery is the first treatment option for most patients [1]. As early BC is curable [2], the number of BC survivors is increasing, and both the short- and long-term sequelae of BC treatment are important. It is essential to carefully balance both the under- and overtreatment of BC. The benefits of a procedure must outweigh the harms, and we must strive for a high quality of life for BC patients during and after treatment. It is necessary to improve clinical algorithms to choose the most optimal treatment strategy while retaining a high level of safety for patients.

The number of nodal metastases is an important prognostic factor for survival in BC, as it is the most obvious distinction between node-positive and -negative BC [1]. Establishing the correct nodal status for BC patients affects decision making regarding the use of adjuvant chemotherapy and radiotherapy in early BC [3]. However, in the era of personalized treatment, the impact of prognostic information from axillary lymph node status on the medical decision-making process is less important than previously, and adjuvant treatment is increasingly tailored toward the biological and genetic features of BC [4].

Axillary ultrasound, the most used imaging modality for nodal assessment, is an unreliable staging modality for BC patients with a low nodal metastatic burden; its negative predictive value (60% to 90%) is not reassuring [5,6,7,8,9]. Different rates of sensitivity and specificity of axillary ultrasound are reported in the literature, ranging from 26% to 94% and from 53% to 98%, respectively [7,8,9,10,11]. Thus, for most BC patients presenting with a clinically and radiologically node-negative axilla at diagnosis, in the quest to reliably stage the axilla, the surgical sentinel-node procedure is routinely used for staging [12]. However, for BC patients aged >70 years with hormone-receptor-positive and human epidermal growth factor receptor 2 (HER2)-negative conditions, sentinel-lymph-node surgery is optional if the patient receives adjuvant endocrine therapy according to ASCO guidelines [12]. However, these guidelines are not implemented in Sweden, and the surgical management of the axilla is the same, independent of age at diagnosis and molecular subtype. In the majority (~80%) of early BC patients undergoing sentinel-node surgery, the procedure identifies only healthy lymph nodes with no metastatic tumor deposits [3,4]. For these patients, the invasive surgical procedure is diagnostic but not therapeutic.

Although there are many benefits of the minor surgical sentinel-node procedure compared to axillary dissection, sentinel-node surgery is associated with short- and long-term side effects. Most importantly, immediate postoperative swelling, paresthesia, arm lymphedema, and self-reported symptoms of arm swelling are documented complications of the sentinel-node procedure [13,14]. In addition, the procedure is time-consuming and requires financial healthcare resources.

We previously developed and published a decision-making tool, the noninvasive lymph node staging (NILS) prediction model, for the prediction of benign lymph nodes in primary BC [15]. The NILS prediction model, which is an artificial neural network (ANN) model, was developed using retrospectively collected variables (including patients, *n* = 800). The NILS prediction model includes ten top-ranked risk variables for nodal status (please refer to Section 2.1), including hormone receptor status but excluding molecular subtype. The aim of this study is to retrospectively validate the NILS prediction model in one temporal and one geographical cohort.

## 2. Materials and Methods

### 2.1. Study Design

**Figure 1:**
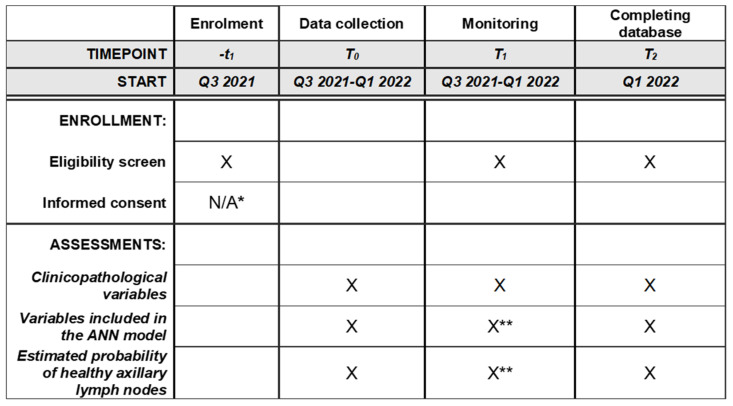
Schedule of enrollment, data collections, and monitoring. * Waived by the ethics committee. ** For a more extensive monitoring schedule for these critical variables, please refer to Supplementary Material S1.

This validation study of the NILS prediction model will be a multicenter, retrospective study based on prospectively collected clinicopathological variables included in patients’ medical charts, mammograms, and pathology reports. We plan to assess the external validity and model validity of the NILS prediction model for healthy lymph nodes and evaluate the discrimination and calibration of the NILS prediction model while following the TRIPOD statement [16]. A schematic schedule of enrollment, data collection, and monitoring is provided in Figure 1. This study aims to validate the NILS prediction model, a previously published ANN-based decision-making tool that is based on retrospectively collected data at Lund University Hospital, Sweden. In agreement with TRIPOD recommendations, the data in this validation study are derived from cohorts different in both time and study sites. Thus, patients who underwent surgery for primary BC in Malmö, Sweden, in 2020 (*n* = 400, a temporal validation cohort) and in Helsingborg, Sweden, between 2019 and 2020 (*n* = 200, a geographical external validation cohort) will be identified by a national registry of cancer diagnoses, treatments, and outcomes (INCA). The data retrieved from clinical files will include age at BC diagnosis, medical history of BC, ultrasound assessment of the axilla, and clinical status of the axilla. Tumor size and localization in the breast, including laterality, multifocality, and mode of tumor detection, will be obtained from imaging assessment reports (mammography, followed by ultrasound assessment in cases of an inconclusive mammographic report). In cases where multifocality is detected by either clinical examination or any imaging modality in this study, the tumor will be considered multifocal. Tumor biology data (estrogen receptor status, progesterone receptor status, HER2 status, molecular and histopathological subtype, vascular invasion, and proliferation index Ki-67) will be obtained using preoperative core needle biopsy. Number of metastatic axillary lymph nodes will be obtained from reports of surgical pathology specimens (sentinel-node surgery and axillary dissection).

The original NILS prediction model for nodal prediction includes ten top-ranked risk variables for nodal status (tumor size, vascular invasion, multifocality, estrogen receptor status, histological type, progesterone receptor status, mode of detection, age, tumor localization in the breast, and Ki-67 positivity) [15]. This validation study will validate the original model, including the aforementioned variables. In addition, important improvements from the early NILS prediction model are that we can validate the algorithm, include predictors that are only preoperatively available (i.e., data on biomarkers and histopathological subtypes from core-biopsy data rather than breast surgical specimens), and estimated tumor size by imaging rather than by histopathology in surgically removed tissue.

**Figure 2:**
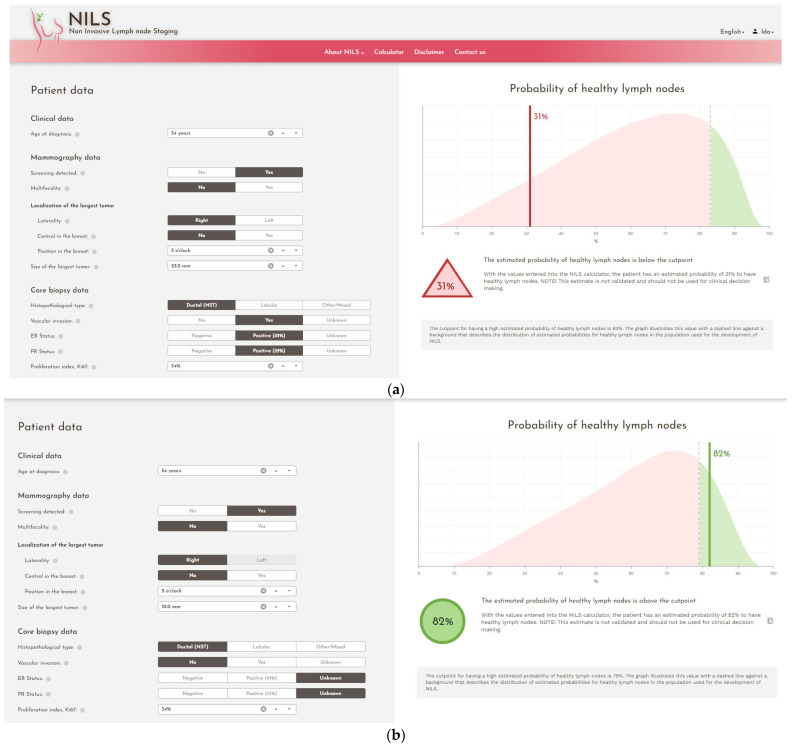
Screenshots from the web interface of the nodal status classifier. (**a**) Data input resulting in an estimated probability of healthy lymph nodes below the cut point, (**b**) data input resulting in an estimated probability of healthy lymph nodes above the cut point.

The decision-making calculator (the NILS prediction model) is derived from an ANN-based model for the prediction of benign lymph nodes at the time of diagnosis in primary BC. The ANN technique can decipher nonlinear and difficult-to-predict associations between risk variables and output. In addition, to handle missing values of important variables such as vascular invasion, an additional ANN model that can predict this value using other available variables was developed. A web-based implementation of the NILS protocol, the nodal status classifier, was developed and tested in a pilot cohort of newly diagnosed BC patients. This web interface is used in this study (Figure 2), providing an estimated probability of healthy lymph nodes for each study participant.

This study protocol was written according to SPIRIT guidelines [17].

### 2.2. Definition of pN0

In this study, pN0 is defined as sampled axillary lymph nodes with no invasive cell clusters of >0.2 mm at the largest diameter (presence of isolated tumor cells, i.e., tumor cell clusters that are ≤0.2 mm at the largest diameter, are considered pN0). 

### 2.3. Ethical Approval

All procedures performed in studies involving human participants were in accordance with the ethical standards of either the institutional or national research committee and with the 1964 Declaration of Helsinki and its later amendments or comparable ethical standards. This study was approved by the Regional Ethics Committee in Lund, Sweden (committee reference number: 2021-00174).

Patients received information about the study through advertisements in the local press, and the patients were allowed to opt out. The ethical committee waived the requirement for informed consent and consent for publication. Upon receiving treatment for BC at the hospitals, the patients provided their consent to register in INCA. The local department for personal data admission at the hospital (KVB Samråd, Region Skåne, Sweden) granted the researchers access, with digital logging, to patients’ digital medical charts. Access to the web calculator is protected by usernames and passwords, which provides permission to authorized users only (physicians, research nurses, and research administrators) from the principal investigator.

### 2.4. Study Population

Each included patient underwent standard diagnostic work-up for the clinical treatment of BC, including a clinical examination, mammogram, ultrasound, and preoperative core biopsy of their tumor. At the time of surgery, the patients underwent standard procedures according to the decision made at the multidisciplinary conference, and surgical specimens comprising the resected breast tumor and axillary lymph nodes were pathologically examined. No extra diagnostic or interventional procedures were performed within this validation study of a retrospective cohort. No results will be reported to the treating physician or study participants.

### 2.5. Participating Centers

Data are retrieved from two independent centers in southern Sweden: Malmö, a university hospital, and Helsingborg, a regional hospital. There were 0.75 million and 0.3 million people in the catchment areas of the breast surgical clinics of Malmö and Helsingborg, respectively.

### 2.6. Power Calculation and Statistical Plan

We estimated that the sample size would be *n* = 300 study participants, and although each validation cohort will be assessed separately, the total number would be *n* = 600. Due to practical reasons (shortage of qualified research personnel at one of the study sites), we will include *n* = 400 from the temporal cohort and *n* = 200 from the geographical cohort. The area under the curve (AUC) for the original prediction model was 0.74 in a development cohort, where two-thirds of the patients were node-negative [15]. Assuming the same proportion of N0 and the same discrimination for future patients, the inclusion of 300 patients is sufficient to obtain 90% probability of reaching an AUC of at least 0.70 in this validation study. This calculation was based on the asymptotic normality of the AUC estimate and simulations that determined the relationship between the sample size and standard error of the AUC estimate. The performance of the ANN decision tool upon validation will be assessed in terms of discrimination (AUC) and calibration (comparison of the observed and predicted event rates of benign axillary nodal status, Hosmer–Lemeshow test). Separate analyses of patients undergoing breast-conserving surgery and mastectomy will be performed. No interim analyses will be performed. Patients will be screened for eligibility according to specified inclusion and exclusion criteria and subsequently included if all criteria are fulfilled. Study participants for whom mandatory variables for the NILS protocol are missing, i.e., variables that the ANN model cannot predict, will be excluded.

### 2.7. Inclusion Criteria

The inclusion criteria are:

1. Accepted study participation (e.g., did not opt out);
2. Female sex;
3. Invasive BC;
4. Negative preoperative assessment of the axilla, clinically and by ultrasound;
5. Scheduled for primary surgery.

### 2.8. Exclusion Criteria

The exclusion criteria are:

1. Did not accept study inclusion (i.e., opted out);
2. Male sex;
3. Children and adolescents (age < 18 years);
4. Previous ipsilateral breast/axillary surgery;
5. Synchronous distant metastases at diagnosis;
6. Previous primary neoadjuvant therapy;
7. Preoperatively verified axillary lymph node metastases by cytology or histology;
8. Positive preoperative assessment of the axilla, clinically and by ultrasound;
9. No surgical axillary staging;
10. Upfront axillary nodal dissection.

### 2.9. Endpoints

#### 2.9.1. Primary Endpoint

The primary endpoint is defined as an axillary nodal status (discrimination, N0 vs. N+) determined from preoperative clinicopathological data from mammograms and core needle biopsies compared to a predictive N status by the algorithm.

#### 2.9.2. Secondary Endpoint

The secondary endpoint is defined as a false-negative rate of a maximum of 10% for the prediction of N0 vs. N+ compared to the histopathological assessment of excised sentinel lymph node(s).

### 2.10. Data Analysis

For secure and trackable data entry, REDCap [18] with audit trail is being used. A comprehensive data-management plan was developed. Only the principal investigator and responsible researchers will be granted access to the final dataset. Personal information of enrolled patients will be collected, shared, and maintained with a high level of caution and according to local and national regulations to protect confidentiality before, during, and after the trial. All collected data will be stored in an encrypted database (REDCap), which will remain in the custody of the principal investigator.

A risk analysis was conducted, and a detailed plan for data monitoring (conducted by an external researcher) was established (Supplementary Material S1).

## 3. Discussion

We present a study protocol for the validation of an ANN model for the prediction of healthy lymph nodes in patients with early BC with clinically and ultrasound-diagnosed node-negative disease (ISRCTN14341750).

The sentinel-node-biopsy technique has a well-established threshold for a false-negative rate of 10% [19,20], and we consider this threshold acceptable in the presented predictive setting of our ANN model. The Swedish Institute of Health Economy conducted a technical report concluding that our prediction model will be cost-saving if its accuracy is consistent with that reported in our previous publication [15].

The optimal management of the axilla in patients with BC is under discussion in clinical and scientific societies. While a normal standard axillary ultrasound examination is insufficient to rule out axillary nodal metastasis [21], improved functional imaging, such as magnetic resonance imaging (MRI) or positron emission tomography/MRI, in preoperative settings provides the same performance as sentinel-node biopsy for excluding axillary lymph node metastases [22,23,24,25,26]. Although these imaging modalities have higher accuracy when determining axillary status, their availability in preoperative settings is not universal, and the presented data are hitherto based on a limited series (Bruckmann et al., *n* = 104; Botsikas et al., *n* = 80; in a review article by Kuijs et al., the largest included study was *n* = 505).

Two randomized controlled trials are investigating the possibility of abstaining from sentinel-node biopsy in patients with BC stage T1 undergoing breast-conserving surgery: the SOUND trial (ClinicalTrials.gov identifier: NCT02167490), which has an estimated data completion date in Q4 2021, and the INSEMA trial, which includes patients with T1–T2 tumors (ClinicalTrials.gov identifier: NCT02466737) and has an estimated data completion date in Q4 2024. The results from these trials will provide qualitative insights into the oncological safety of omitting sentinel-node surgery.

Data supporting the impact of the pathological staging of the axilla on adjuvant systemic treatment recommendations are limited to luminal A-like tumors; thus, the need to detect axillary lymph node metastasis in early BC is of less importance. In a study by van Roozendaal et al., the decision to recommend adjuvant systemic treatment changed in only 1% of the patients due to a pathological lymph node status when using Adjuvant! Online [27], and a report of *n* = 1001 patients included in the INSEMA trial showed that tumor biological parameters alone guided systemic treatment decisions in 99% of the patients [4].

In the NILS prediction model, hormonal status and Ki67, but not molecular subtype, are included. In the initial development of the model, presented by Dihge et al., a strong correlation was found between grade and Ki67 [15]. Grade was excluded and Ki67 was retained in the final model to minimize the risk of collinearity [15]. In addition, HER2 was not identified as a predictor of nodal status and was not included.

Many nomograms and ANN models have been developed for predicting nodal metastasis, or the lack thereof, with [28,29,30,31,32,33,34,35,36] or without imaging [37,38,39,40]. The discrimination of clinical nomograms including clinicopathological data only is equivalent to the predictions made by the NILS prediction model (area under the curve (AUC) 0.73–0.75 [39,40]), whereas those incorporating radiomic features present an AUC slightly above 0.90 [30,31,32,34]. However, the ANN model that this study aims to validate is a promising tool in the clinic because the input variables are routinely available, no extra imaging besides clinical work-up (i.e., mammography and axillary ultrasound) is necessary, and the web interface is user-friendly.

### 3.1. Significance

Internationally, there are numerous artificial intelligence projects involving the noninvasive identification of N0 BC. The NILS protocol uses easily accessible clinicopathological variables, including mammography data, and, in contrast to many other algorithms, does not require medical imaging data for machine learning analysis. We present a robust validation study of the NILS prediction model based on two external cohorts. If the accuracy and discrimination reach a satisfactory level, our prediction tool (the NILS protocol) can be implemented and would assist medical professionals and BC patients in shared decision making on omitting sentinel-node biopsy in patients predicted to be node-negative. In the future, the use of the NILS protocol may save healthcare resources and reduce both costs and unbeneficial side effects. In addition, the NILS prediction model and our presented validation study may prompt future studies of nodal metastases of malignancies in other organs; thus, it may have implications beyond BC. 

### 3.2. Current Status

Clinicopathological data are being retrieved from electronic medical charts and pathology reports along with the output from the web application for the ANN. Specially trained research nurses and research administrators with many years of experience in data management are responsible for data entry. Data entry began in Q3 2021 and will be completed in Q1 2022.

## Data Availability

This is not applicable to the study protocol. Following the enrollment of participants, the raw datasets are kept with the corresponding author and available upon reasonable request due to restrictions, such as privacy and ethical restrictions. The data are not publicly available because of these restrictions. The study results will be made public through a peer-reviewed publication in an international journal. The full protocol, participant-level dataset, and statistical code will not be publicly available because of privacy and ethical restrictions.

## Supplementary Materials

The following supporting information can be downloaded at: https://www.mdpi.com/article/10.3390/diagnostics12030582/s1, S1: Data monitoring
